# Scalable Combinatorial Tools for Health Disparities Research

**DOI:** 10.3390/ijerph111010419

**Published:** 2014-10-10

**Authors:** Michael A. Langston, Robert S. Levine, Barbara J. Kilbourne, Gary L. Rogers, Anne D. Kershenbaum, Suzanne H. Baktash, Steven S. Coughlin, Arnold M. Saxton, Vincent K. Agboto, Darryl B. Hood, Maureen Y. Litchveld, Tonny J. Oyana, Patricia Matthews-Juarez, Paul D. Juarez

**Affiliations:** 1 Department of Electrical Engineering and Computer Science, University of Tennessee, Knoxville, TN 37996, USA; E-Mail: sbaktash@utk.edu; 2 Department of Family and Community Medicine, Meharry Medical College, Nashville, TN 37208, USA; E-Mails: rlevine@mmc.edu (R.S.L.); bkilbourne@mmc.edu (B.J.K.); vagboto@mmc.edu (V.K.A.); 3 National Institute for Computational Sciences, Oak Ridge National Laboratory, Oak Ridge, TN 37831, USA; E-Mail: grogers3@utk.edu; 4 Department of Public Health, University of Tennessee, Knoxville, TN 37996, USA; E-Mail: akershen@utk.edu; 5 Department of Epidemiology, Emory University, Atlanta, GA 30322, USA; E-Mail: stevecatlanta@aol.com; 6 Department of Animal Science, Institute of Agriculture, University of Tennessee, Knoxville, TN 37996, USA; E-Mail: asaxton@utk.edu; 7 Division of Environmental Health Sciences, College of Public Health, Ohio State University, Columbus, OH 43210, USA; E-Mail: dhood@cph.osu.edu; 8 Department of Global Environmental Health Sciences, Tulane University, New Orleans, LA 70112, USA; E-Mail: mlichtve@tulane.edu; 9 Research Center on Health Disparities, Equity, and the Exposome, University of Tennessee Health Science Center, Memphis, TN 38163, USA; E-Mails: toyana@uthsc.edu (T.J.O.); pmatthe3@uthsc.edu (P.M.-J.); pjuarez@uthsc.edu (P.D.J.)

**Keywords:** combinatorial algorithms, data science, graph theoretical techniques, health disparities research, heterogeneous data analysis, high performance computing, public health exposome, relevance networks, scalable computation

## Abstract

Despite staggering investments made in unraveling the human genome, current estimates suggest that as much as 90% of the variance in cancer and chronic diseases can be attributed to factors outside an individual’s genetic endowment, particularly to environmental exposures experienced across his or her life course. New analytical approaches are clearly required as investigators turn to complicated systems theory and ecological, place-based and life-history perspectives in order to understand more clearly the relationships between social determinants, environmental exposures and health disparities. While traditional data analysis techniques remain foundational to health disparities research, they are easily overwhelmed by the ever-increasing size and heterogeneity of available data needed to illuminate latent gene x environment interactions. This has prompted the adaptation and application of scalable combinatorial methods, many from genome science research, to the study of population health. Most of these powerful tools are algorithmically sophisticated, highly automated and mathematically abstract. Their utility motivates the main theme of this paper, which is to describe real applications of innovative transdisciplinary models and analyses in an effort to help move the research community closer toward identifying the causal mechanisms and associated environmental contexts underlying health disparities. The public health exposome is used as a contemporary focus for addressing the complex nature of this subject.

## 1. Background

Research on health disparities in racial/ethnic and disadvantaged groups has received increased attention in recent years. More and better data, new analysis techniques, and focused research programs have helped increase our understanding of issues and potential solutions. One such program, initiated by the authors of this exposition, is the National Health Disparities Research Center of Excellence (HDRCOE), begun over a decade ago at Meharry Medical College in Nashville, Tennessee. Supported by the National Institute on Minority Health and Health Disparities, the HDRCOE is an inter-institutional, inter-disciplinary center designed to study the complex relationships between human factors, community context and macro social forces that may lead to health disparities.

A newly established center, with which this paper’s authors are also intrinsically affiliated, is the Research Center on Health Disparities, Equity, and the Exposome (RCHDEE) at the University of Tennessee Health Science Center in Memphis, Tennessee. The RCHDEE is a collaboration that brings together academic and community partners to conduct health disparities research in an effort to promote healthy neighborhoods and help eliminate disparities.

The public health exposome model [1] identifies environmental exposures in four broad domains: natural, built, social, and policy. This model is used to guide the RCHDEE’s research agenda. It builds on the exposome paradigm [2], and is aimed at describing the effects of multiple and cumulative environmental exposures from conception to death on population health outcomes using a life stage approach. The RCHDEE brings together a transdisciplinary team of investigators with training in traditional epidemiologic and statistical methods with those proficient in the use of advanced computational, multi-level, and spatial models and analytics [3].

A complete understanding of the mechanisms through which multiple and cumulative environmental exposures across the life span can affect individual and population health is not yet attainable. Nevertheless, the public health exposome model can help generate hypotheses and interpret ways in which population health outcomes are the combined product of the presence or absence of individual and ecological risk and protective influences. These may include social determinants, life events, epigenetics, toxic exposures, social networks, access to healthcare, and numerous other subtle and under-appreciated factors.

An amalgam of diverse scientific techniques are described in this paper: computer science, mathematics and statistics join forces with data interpretation and domain knowledge to elucidate both known and previously unrecognized variable relationships, and to generate testable hypotheses on an unprecedented scale. Pioneering graph theoretical methods and their application to modern health disparities research are employed. Practical use is made of lessons learned over the last two decades in the analysis of high throughput biological data. While standard techniques can scrutinize at most a handful of parameters for obvious dependencies, combinatorial methods are able to extract latent signal from a sea of even only modest correlations spread across an entire spectrum of available variables. A prototypical toolchain and illustrative examples are also presented.

This work can be placed in the context of health science research transformations or paradigm shifts [4]. Urged by the National Cancer Institute and the National Institute on Environmental Health Sciences, the scientific community has developed thematic recommendations [5] for health disparities investigations that include transdisciplinary knowledge integration, data sharing, and an expanded use of quantitative methods to include multilevel analyses, spatial analysis, and the utilization of so-called “big data.” In a companion paper [6] we discuss many of these issues in depth.

## 2. Introduction

In an all-too-common scenario, epidemiologists, social scientists and other research personnel labor over data reported at an aggregate level and categorized by location (say, for example, boroughs, counties and parishes). Yet despite the burgeoning richness of available information, only a limited number of items are considered. Regression may be used to estimate the individual effect of a single explanatory variable on an outcome. This in turn may seriously overweight the presumed significance of that one variable, and discount the importance of complex and convoluted relationships that are often hidden within the data [7]. The virtual avalanche of explanatory variables now readily available renders traditional methods and models such as these inadequate for modern relational analysis.

Scalable computational techniques (those that remain practical and efficient when applied to ever larger problems and/or platforms) are needed to handle the gigantic volumes of inhomogeneous data now streaming on line, and to identify more accurately the full range of variables relevant to outcomes. The techniques we describe in this paper represent just such an approach. Born of our long-standing work in graph algorithms, parameterized computing and complexity theory, we achieve scalability through mathematical abstraction, eschew bias by assuming no prior knowledge, gain asymptotic efficiency with emergent algorithmic techniques, and winnow out all but the most likely feasible solutions without sacrificing an effective examination of all explanatory variables. Our approach applies powerful graph theoretical algorithms to scan the entire solution space and organize explanatory variables into tightly inter-correlated groups. It then prioritizes those groups most closely related to the outcome under study, so that their significance can be confirmed or refuted with widely available standard, traditional analytical methods.

By harnessing state-of-the-art tools such as these, social behavioral scientists are able to realize the increasingly popular promise of “big data.” Compelling hypotheses can now be the output of the discovery process, not just the input to experimental design. What’s more, the data can speak for itself, in that complex and subtle relationships can be discerned without pre-conceived notions, long before plausibility much less causality can be established. This approach provides the opportunity to uncover relationships we might otherwise never even have thought to consider, perhaps because we are biased by our previous training and experience, or because we are intimidated by the sheer size of the solution space.

**Figure 1:**
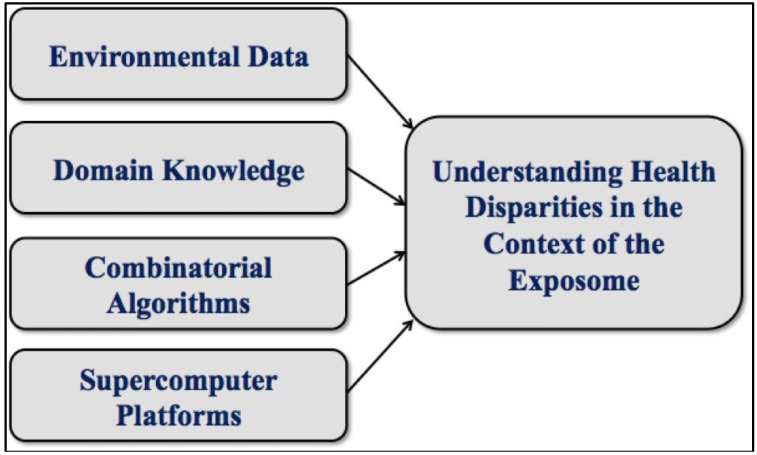
A confluence of resources and technologies.

Powerful new analytical techniques such as those we will describe have the potential to revolutionize and help automate various important aspects of health disparities research, just as they have in computational molecular biology. Indeed, much of what we have learned is based on our many years spent designing algorithms for the analysis of high throughput biological data. Today’s health disparities research, like genomic science before it, benefits from the timely confluence of broadening resources and emerging technologies. See Figure 1.

Parallels between high throughput biological data analysis and health disparities research are worthy of discussion. One property common to both fields is found in the discovery process. The search for signature disease genes (those with allelic variants thought to be primarily responsible for disease) has largely been a failure. Single gene predispositions certainly exist, but it turns out that the genetics underlying most diseases appears to be overwhelmingly *complex* (non-Mendelian) and thus due, not to a single DNA locus, but instead to numerous loci scattered throughout the genome. Moreover, some of these sites lie within cis-regulatory elements, others seem to effect the expression of distal genes, and still others have no discernable effect on coding regions at all. It seems, therefore, that three-dimensional structure, not mere nucleotide sequence, is critical. In addition to all this, there are epigenetic, environmental and numerous other factors that come into play. The point is that genetic actors in disease often work within complicated, poorly understood, and highly nonlinear relationships. Analogies to actors in the social sciences are surely manifest to almost anyone who has tried to unravel complex relationships in the health disparities domain. This observation prompts a need for powerful, scalable computational tools, not unlike those developed to study high throughput, high dimensional biological data, that can extract not just one or a few but all possible combinations of interrelated factors.

The parallel continues as we grapple with difficulties posed by known but accepted shortcomings in data quality. Data in both fields are often unstructured, noisy, mis-measured, mis-labeled and mis-aligned. Missing values and inconsistent scales further compound the problem. A huge assortment of combinatorial and statistical strategies has been developed for dealing with these sorts of issues in modern biological science. We have learned how to modify and leverage these strategies in applications to health disparities research, rather than re-inventing them or, worse yet, continuing on the path of traditional low-throughput analysis as if no better methods were available.

Finally, and most foundationally, both fields focus on variables, items that are measured. In biology a variable may mean a gene, a transcript, a protein, a metabolite or some other omics unit. In health disparities research, we are more likely to focus on variables closely associated with social determinants, such as employment, education, ethnicity, access to healthcare and so forth. Both fields also employ correlation. Despite obvious shortcomings, for example, the unfortunate and ceaseless confusion with causation, correlation is fundamental to quantifying relational strength. We therefore find that, just as genes may be highly correlated by their expression profiles over a set of stimuli, variables associated with social health determinants may be highly correlated by the manner in which they vary across spatial or temporal units. Classic biological analogies include “relevance networks” and “guilt by association.” See, for example [8,9]. In the sequel, we will refer to variables as vertex labels and correlations as edge weights in graphs constructed from raw data in preparation for analysis.

## 3. Graph Theoretical Utility

At this point one might ask: what advantages does graph theory have, and how does it scale to immense, otherwise recalcitrant problems? The systematic study of graph theory can be traced back nearly 300 years, at least as far back as the seminal work of Euler on crossing the seven bridges over the Pregel River in Königsberg, Prussia [10]. Since that time, graph theory and graph algorithms have grown to become mainstream subjects in mathematics, computer science, operations research and related disciplines. Well-known sample problems and methods include graph coloring, planarity testing, graph Hamiltonicity and network flow, to name just a few. Today, graphs are used to model everything from electrical circuits, to chemical compounds, to biological pathways, to transportation and social networks, and even to the so-called information superhighway.

Graph theoretical algorithms focus mainly on connectivity and structure. They generally come with no preconceptions, semantics or assumptions about distance or dimensionality. They also are flexible. Once a graph is created, a wide assortment of metrics can be applied. Furthermore, in many applications, most notably the ones we discuss here, we can employ novel computational strategies and high performance platforms to overcome what were until recently formidable computational bottlenecks.

While terms such as “graph” and “network” are sometimes used interchangeably, we caution the reader that the former has a precise mathematical definition, while the latter can take on a variety of meanings. This is particularly relevant in the present context, because some of the products we produce with graph theoretical algorithms are dense subgraphs, each of which may then be viewed as a network of variables in social science and epidemiological applications.

## 4. Graph Algorithmic Methods

Today, the study of graph theory and graph algorithms has expanded from computer science and discrete mathematics to nearly all scientific fields of endeavor. In this treatment, as much as possible, we will try to avoid highly technical definitions and complex notation. We refer the interested reader to the wide assortment of introductory references that are available for this subject. See, for example [11].

**Figure 2:**
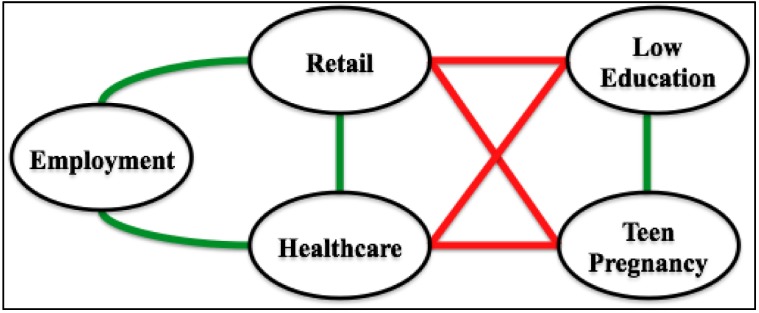
A sample graph fragment.

Unless otherwise stated, all graphs we consider are finite, simple and undirected. Such a graph contains vertices, which usually denote items of potential interest, and edges, which represent relationships between selected pairs of vertices. There are numerous ways to construct such a graph. In the context of health disparities research, for instance, vertices may represent numeric data, both integer and real. Possible examples include median age, average income, employment percentage and immunization rate. Vertices may also represent categorical data. Examples include employment type, predominant ethnicity, healthcare availability, and primary cause of mortality/morbidity. An edge, in contrast, generally denotes some sort of commonality between the two vertices it connects. Such an edge can be weighted in order to specify the strength of this commonality. For example, one might expect to find a highly weighted edge between a vertex that represents proximity between very poor and middle class neighborhoods and another vertex that represents rates of violent crime, because in many communities these two measures may seem closely (and positively) related [12]. These notions are illustrated in Figure 2, which depicts a 5-vertex subgraph of a 596-vertex graph constructed from data taken over Gulf Coast counties and parishes whose health care systems were severely affected by hurricane Katrina in 2005. Only correlations with R > 0.25 are shown, with positive relationships indicated by green edges and negative relationships depicted in red. 

Structure is often key to unraveling complex graph theoretical connections and dependencies. Literally hundreds of structural graph metrics can be defined. One of the most important and widely studied of these is density. The densest subgraph on *k* vertices contains all *k(k−1)/2* possible edges, and is called a *clique*. Cliques come in many forms. There are maximum cliques, maximal cliques, bicliques, paracliques, clique communities and so forth. See, for example [13].

Clique optimizations are almost always computationally intractable (technically they are *NP*-hard [14]). For graphs of even moderate size, therefore, successful clique-centric approaches generally require some form of cutting-edge mathematical techniques, sophisticated algorithms, efficient implementations and high performance realizations. A good example, and one relevant to the tools we describe here, is recent work on fixed parameter tractability and its application to vertex cover as a complementary dual for solving clique [15]. (A problem of size *n*, parameterized by *k*, is fixed parameter tractable if it can be decided in *O(f(k)n^c^)* time, where *f* is an arbitrary function and *c* is a constant independent of *n* and *k*).

## 5. Supporting Technologies

An assortment of auxiliary technologies may be helpful in order to apply graph theoretical algorithms to problems in health disparities research. These technologies may be loosely classified as pre- and post-processing. The former is usually required in order to prepare a graph for analysis; the latter is applied to the results of graph theoretical evaluation and is oftentimes optional.

In pre-processing, we seek to build a (typically enormous) graph from raw data so that its structure preserves important relational information. We can then apply highly-scalable graph theoretical algorithms in order to extract (typically small) subgraphs based on density or some other relevant metric. Data requirements and measurement/reading multiplicities are frequently demanding, in large part because we need enough information to compute edge weights. Absent other information, normalization and correlation are the usual weapons of choice for this. Pearson product-moment coefficients are more or less the gold standard. Based upon data quality, the likelihood of outliers, project objectives and other factors, however, we may instead derive coefficients using other measures including mutual information, Spearman's rank order, and sometimes even Euclidean distance. From these computations we can begin to harvest the power of abstraction, by reducing raw data to a (symmetric) correlation matrix, *M*. The correlation coefficient relating variable *i* to variable *j* is found at matrix location *M(i,j)*. *M* can thus be viewed as a weighted adjacency matrix for the complete correlation graph, *G*, whose rows (and columns) represent vertices and whose entries denote edgeweights.

Pre-processing also necessitates a thresholding operation, because graph theoretical algorithms generally (though not always) perform their tasks on unweighted objects. Thus, the goal is to select within some predefined coefficient range a threshold, *t*, so that any edge with weight less than *t* is discarded, and weights on remaining edges are ignored. (Absolute correlation values are usually employed, because positive and negative relationships tend to be of roughly equal significance). The result is a finite, simple, undirected and unweighted graph that, depending on the choice of *t*, typically has some but not all possible edges. Thresholding is highly domain-dependent, and frequently there is no single right or best choice for *t*. In our own work in computational biology, for example, we have used spectral methods, clique-centric inflections and even domain knowledge to select thresholds, often with the aid of extensive repositories of ontological characterizations [16,17]. With no similar knowledge base in health disparities research, we are left largely to empirical methods for threshold selection.

Post-processing, though not strictly required, is often highly desirable. Here the goal is to determine whether there is independent, orthogonal, *in silico* confirmation of results. Rather than focus on global structure, the objective now is to estimate the magnitude and independence of associations between specific health outcomes and the factors captured by dense subgraphs identified through computational analysis. As always, we must neither confuse raw data with fact, nor correlation with causation. When two or more computational techniques produce the same results, however, we tend to have more confidence in their validity. We may also be much more inclined to invest the time and resources required for further validation. By this we mean validation outside and apart from any dependence on existing data or the use of computation. Such validation has traditionally been termed “wet lab” work in biology. In the health disparities setting, instead, we may seek community involvement, intervention or patient centered outcomes.

Post-processing can be performed with a variety of traditional techniques. It does not require novel mathematical methods or extreme algorithmic sophistication, because we have already applied scalable graph theoretical tools to examine the entire solution space and produce highly distilled sets of pairwise adjacent variables. Post-processing, therefore, is not expected to produce results on this scale. It needs only to confirm or refute them. Common validation approaches include multiple regression, confirmatory factor analysis and structural equation modeling, which can be used to assemble latent constructs in an effort to explain variance. In recent years, mediation analysis has also become very popular. Alternately, Bayesian methods can be employed to model unknown distributions and impute directionality. A relevant example is Bayesian multi-level analysis, in which prior information is coupled with the notion that biological, psychological, environmental, social and other processes that may influence human health can occur at many levels, some of which may be nested within others. R, SAS and other widely-available software packages are available for post-processing techniques such as these.

## 6. Analytical Toolchain

In order to apply methods like those just described, we begin with the collection of raw data and invoke a variety of pre-processing steps, using domain expertise to filter and segregate it as necessary depending on the application. Multilevel modeling, dimension reduction and other data manipulations are possible at this stage. We next apply various normalizations, and eliminate from consideration any variables with near zero variation. At this time we are able to compute correlation matrices, and apply thresholding with spectral or other methods to generate unweighted graphs. In such a graph, vertices represent variables of interest, and edges denote significant variable-variable correlations.

We are now ready to apply graph theoretical algorithms and, as needed, high performance computation. To accomplish this, we will extract cliques, paracliques and other dense subgraphs for further study. We often handle graphs in which vertices number in the hundreds, thousands and even millions. Additionally, although subgraph sizes are a function of the application and the threshold chosen, in practice we tend to produce subgraphs with but a few tens of vertices. Each vertex within such a subgraph represents a variable that is highly correlated with all or almost all others represented in the same subgraph. Some of these variable sets may be easily recognized by health scientists, while connections within others may not have been previously identified in the literature.

At this time, each subgraph (set of variables) constitutes a putative result ready for post-processing and detailed scrutiny with a variety of exhaustive, but not scalable, orthogonal methods. We employ traditional tools to test covariance structures, estimate alpha reliabilities, and create credible constructs with standardized metrics. Construct variables can then be included in further structural equation models to test the validity of the construct toward predicting desired outcomes. Occasionally, a single variable is closely associated with an outcome of interest. More often, relationships are complex, and two or more variables are required. Constructs can also be saved, and used later in Bayesian and other alternative analyses. Coupled once again with domain knowledge, any construct so confirmed now becomes a testable hypothesis, which may be explored in various ways, for example, with new data or by on-site validation studies.

**Figure 3:**
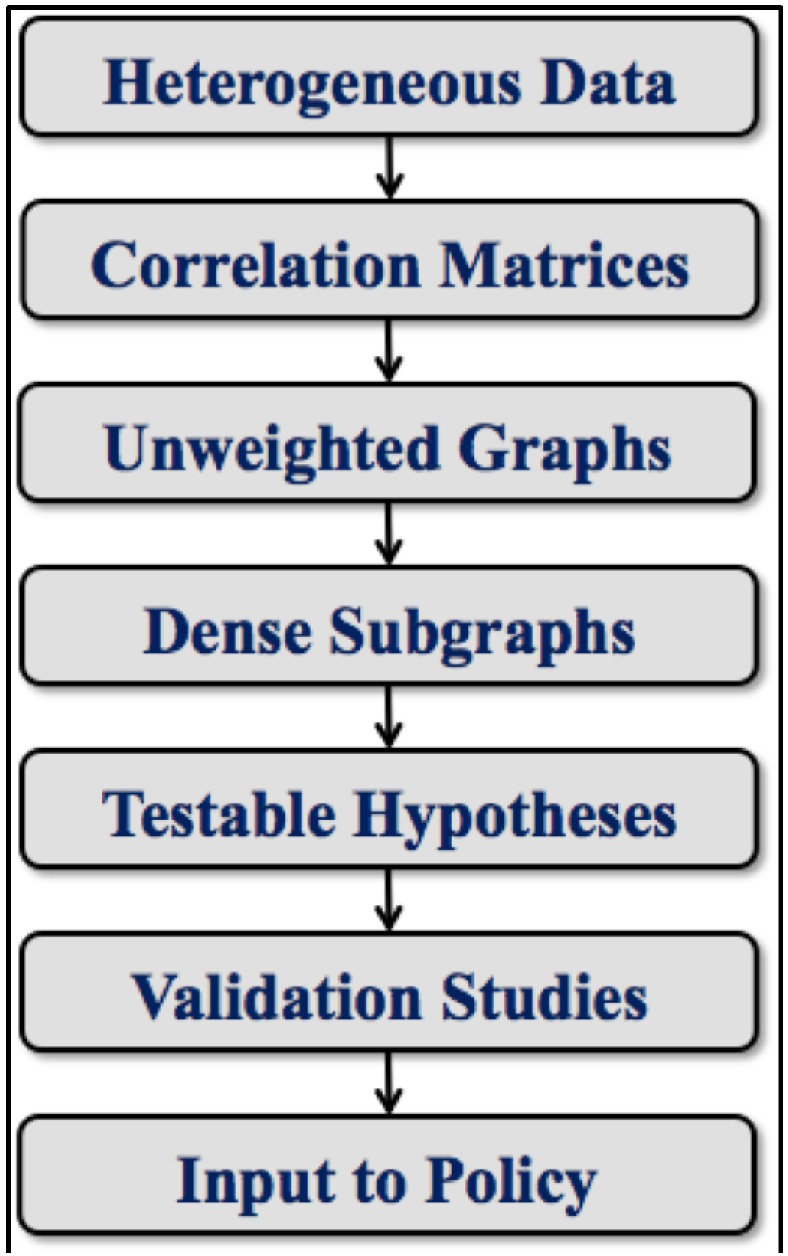
A simplified toolchain for health disparities data analysis.

Thus, we have moved the analysis from raw data capture all the way through the pipeline to “boots on the ground” investigations. Conclusions drawn at this point make potential inputs to changes in policy at local, regional and, in principle, even the national level. An overall schematic of this process is provided by the toolchain depicted in Figure 3. The workflow illustrated there is of course an oversimplification. Numerous dynamic details are suppressed, for example, arrows to suggest feedback and re-tuning that may occur at every level. We note again how this general approach is adopted from our prior work in computational biology and the study of relevance networks. We have mainly modified it to address the needs of health disparities research. The only other investigation we have come across that uses an approach to exposome analysis roughly analogous to ours is described in an interesting but as-yet unpublished arXiv entry about work done in France focused on occupational health [18]. Good ideas tend to proliferate. We would not be surprised to find other examples.

## 7. Refinements and Variations

A great many refinements and variations on this general line of investigation are possible. We mention only a few:

- *Differential Analysis* is frequently added to the computational mix. Vertex, edge and topological differences can be highly informative, particularly in case/control settings [19,20].
- *Feature Selection* can aid in early dimension reduction by eliminating irrelevant variables and bringing focus to the most important regions of the input space [21]. In the extreme case, one might even eliminate all variables or readings not associated with a single outcome.
- *Partial Correlations* [22], Shrinkage [23] and/or Gaussian Graphical Models [24] are sometimes employed, especially when there are only a limited number of readings per variable.
- *Fuzzy Computations* are often preferred, in an effort to counteract or at least ameliorate the effects of noise. Examples include soft thresholding and near-clique extractions [25].
- *Subgraph Overlap* may increase fidelity. By tuning our codes to provide this feature, a vertex may reside in more than one subgraph, just as a variable may be involved in more than one relationship [26].
- *Domain Knowledge*, usually invoked only at toolchain extremes, can sometimes be applied midstream. Subgraphs can be anchored at variables of established significance. Thresholds can be set using specific knowledge of variable-variable interaction strengths.

## 8. Exemplars

We now present a set of exemplars, or case studies, to illustrate the application of algorithmic techniques such as those we have just described. We focus on prematurity, longevity and lung cancer, three highly contrasting and representative open problems in health disparities research. These offerings are in no way intended to serve as finished products. We seek instead to highlight and explain how graph theoretical tools lift our efforts to a new level, and to showcase actual work in progress. For the reader’s convenience, we summarize these applications in Table 1.

**Table 1:** Exemplars.

Exemplar	Prematurity	Longevity	Lung Cancer
Study Design	One Case	Two Case	Eight Case
Selection Basis	Population	Mortality	Race, Sex, Mortality
Refinement	------	------	ANOVA
Hypothesis Generation	Stand-Alone	Differential	Differential
Traditional Verification	Bayesian Analysis	------	------

As is customary, we employ the term “county” to mean a county or a county equivalent, such as a borough or parish. In the accompanying figures, vertex labels are kept short for readability. Abbreviated data dictionaries listing label meanings can be found in the Appendix.

### 8.1. Prematurity

**Figure 4:**
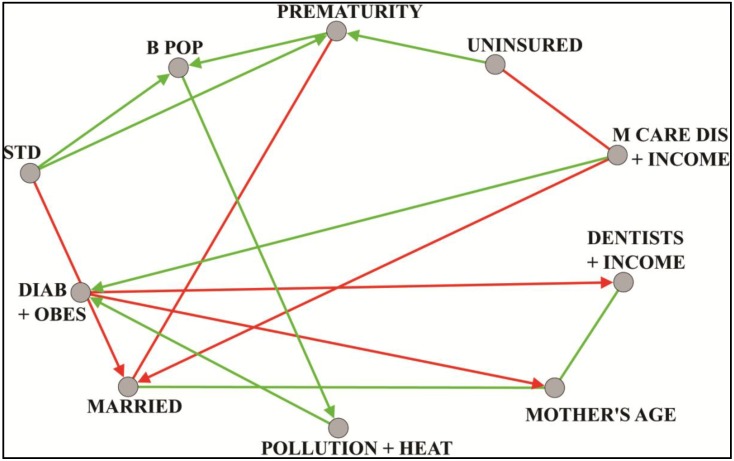
Unsupervised Bayesian network of latent factors derived from paracliques computed in prematurity analysis.

Preterm birth, defined as birth at less than 37 completed weeks of gestation, is a significant risk factor for infant death [27,28]. Some two-thirds of all such deaths occur among infants born preterm. There are notable disparities, however, in premature birth rates, as has been documented by the Centers for Disease Control and Prevention (CDC) and the National Bureau on Health Statistics. Black or African-American women have higher preterm rates compared to non-Hispanic white populations [29,30,31]. We therefore studied county birth rate variations, and looked at potential predictors to identify possible means of prevention. Birth rate was measured using the CDC Wonder national natality files over the period 2003–2011, in counties with populations of 100,000 persons or more. A county premature birth rate was calculated as the number of live singleton births with 24–33 weeks gestation, as a proportion of live singleton births with 24–47 weeks gestation. A total of 590 explanatory variables were used, each denoting a vertex in the correlation graph, and each representing some indicator taken from the economic, health care, physical or social environment. Examples include mean mother’s age, proportion black/African-American, per capita income and adult obesity rate. Edges between vertices were weighted with Pearson correlation coefficients. Thresholding was performed at *t* = 0.61 using absolute values and spectral methods [17]. Dense subgraphs were then extracted with the paraclique algorithm [25]. The median correlation between prematurity outcome and each paraclique was calculated. Those paracliques with correlations at or above 0.38 were chosen for further study. From these, exploratory factor analysis was used to identify latent factors. Regression, with backwards elimination and spatial autocorrelation, was next performed to select those factors most predictive of premature birth. Finally, a Bayesian network was constructed to hypothesize directional relationships (structural equation modeling, for example, would have been an alternative approach). An illustrative hypothesis links prematurity to the rate of sexually transmitted disease (STD). See Figure 4, whose vertex label definitions can be found in Table A1 in the appendix. We should add that any hypothesis so generated must take into account possible data limitations. For example, STD rates may be affected by differential reporting across socio-economic groups and differential practices across counties. Additional work focused on prematurity can be found in [32].

### 8.2. Longevity

**Figure 5:**
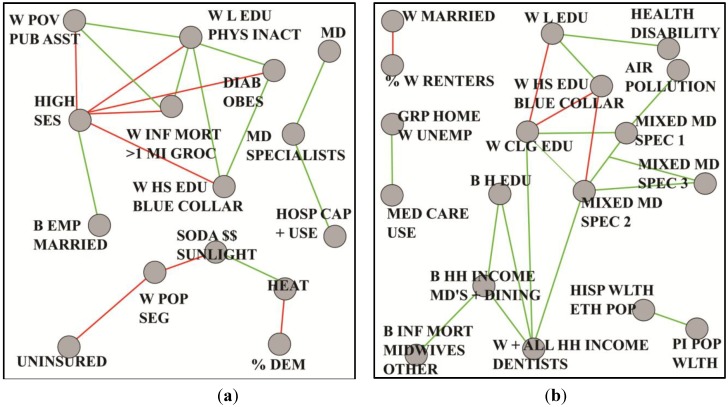
Coarsened graphs based on mortality among black/African-American men, in two sets of 39 counties each, where age-adjusted (25–64 years) 1999–2010 all-cause black mortality is (**a**) lower than the corresponding average for white men and (**b**) highest overall.

We have previously observed [33] that during 1999–2007, counties with significantly lower overall age-adjusted mortality among black or African-American men ages 25 to 64 years, than that found for whites, had greater proximity to military bases and higher percentages of military veterans. There was also less poverty, higher *per capita* income and greater educational attainment. The present analyses continue this inquiry, using the previously reported methods to obtain updated data. Among all U.S. sites with reliable mortality rates, we identified two sets of 39 counties meeting the aforementioned criterion for years 1999–2010. The demographic profile of these counties as well as proximity to military bases and persistence of relatively low mortality over the past 30 years was maintained. We employed a graph theoretical approach as previously described to compare these two sets of counties. A Pearson correlation graph was constructed for each set. Absolute thresholds were computed with spectral methods, resulting in *t* = 0.74 for low mortality and *t* = 0.84 for high. Paracliques were then extracted and used as nodes in a pair of coarsened graphs, one for each set of counties. Inter-paraclique edge weights were derived using median correlations and majority polarity. Finally, the coarsened graphs were thresholded to match relative densities. See Figure 5, whose vertex label definitions can be found in Table A2 and Table A3 in the Appendix. While a more detailed discussion of these findings is beyond the scope of this paper, initial inspection reveals several interesting differences in the structure of the two sets of counties that would have been difficult to detect without this combinatorial approach. For example, in Figure 5a we discover that a prominent feature of the low mortality counties is socioeconomic status (SES), which is negatively correlated with important individual and community aspects such as infant mortality, physical inactivity, diabetes and obesity.

In contrast, Figure 5b shows that the high mortality counties exhibit a network of special medical services that stand in contrast to the absence of such services in the low mortality counties. Ongoing analyses [34] include multivariate regression and structural equation modeling, coupled with domain knowledge pertaining to plausibility and other factors to generate new hypotheses about contextual population factors and mortality among young and middle-age black or African-American men. A next logical step will ideally involve an on-the-ground analytic epidemiologic investigation to test these hypotheses.

### 8.3. Lung Cancer

**Figure 6:**
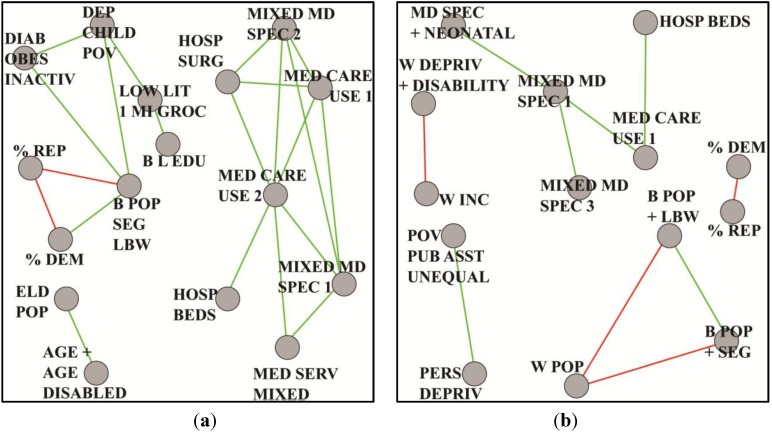
Coarsened graphs based on lung cancer mortality, preselected for males and high disease incidence, where (**a**) rates are for blacks/African-Americans and (**b**) rates are for whites.

The National Institute on Minority Health and Health Disparities has designated lung cancer as an investigative priority due to disproportionately higher incidence and mortality rates among blacks or African-Americans than among other ethnic groups [35,36,37]. We began this study with a county-level focus based on race, gender and age-adjusted lung cancer mortality for individuals aged 25 years and older. These three criteria meant that we could, in principle, subdivide the analysis into eight disjoint groups, each differing from the others in at least one dimension. Pre-processing with ANOVA revealed that high and low mortality groups were significantly different from one another. From this observation, plus known higher incidence and mortality rates among males, we elected to reduce dimensionality by limiting our attention to males and high cancer rates. Thus, gender and mortality were trumped by race, reducing the analysis to just two classes of counties, which we term black male high (BMH) and white male high (WMH). Statistical and graph theoretical tools were applied as previously described. Pearson correlations and an absolute threshold of *t* = 0.60 produced a pair of graphs, from which we extracted paracliques. Confirmatory factor analysis was next used to construct latent variables, each containing at least four variables, for every paraclique. Tau equivalent models were applied when nodes contained fewer than four variables [38]. These latent factors were then used as nodes in coarsened graphs. Inter-factor edge weights were computed with median correlations and majority polarity. Coarsened graphs were thresholded to match relative densities. See Figure 6, whose vertex label definitions can be found in Table A4 and Table A5 in the appendix.

These graphs provide insight into possible underlying bases for health disparities. In Figure 6a, for example, we see in the BMH community a much greater connectivity in health care capacity, services and use. This, combined with connections from obesity, diabetes and inactivity to measures relevant to weak socioeconomic status, warrants further research into determining whether blacks or African-Americans are in poorer overall health than whites. On the other hand, Figure 6b suggests a minimality of structure, few indicators of health care capacity and use, and little evidence of co-morbidities. Persistent deprivation and public assistance predominate. Continuing analyses [39] are taking us in several directions. The incidence of lung cancer, for example, is clearly associated with smoking. Nevertheless, its differential role in mortality across groups is less clear. Smoking variables did not appear in paracliques, because they did not correlate with other paraclique variables at threshold levels. An anchored approach finds numerous variables correlated with smoking at 0.30. Connections are therefore present. They are significant and pervasive. Yet they are too subtle to rise to a level high enough to make them obvious in the context of mortality. Factors associated with access by blacks or African-Americans to medical specialists, hospital admissions and capacity, as well as education and existing co-morbidities serve as important components of future hypotheses on differential lung cancer mortality.

## 9. Limitations

The data used in this study are subject to several well-documented limitations that may arise from various sources, including death certificates [40], medical records [41,42,43], public information pertaining to health care resources [44], the US Census [45], environmental sampling [46,47], and the use of population-based socio-demographic data as a surrogate for individual information [48,49]. Additionally, differing temporal units are sometimes assigned to both predictor and outcome variables. One must also be wary of ecological fallacy [50], in that these data, both health outcomes and environmental exposures, are largely ecological and/or spatial, and not based on specific individuals.

To illustrate, we observe that county-level data used for the study of environmental pollutants does not reflect exposures of individual residents. This may occur for any number of reasons, as examples, the transfer and distribution of pollutants from one geographic region to another by wind or other forces, the fact that an individual’s place of residence may not be where he or she works, and the recognition that current illnesses may reflect exposures from an individual’s past, even including exposures in utero (which might in turn reflect, in part, residua of lifetime maternal and/or paternal exposures). Neither is it absolutely certain that average exposures measured at the county-equivalent level will reflect the exposures of individuals with health outcomes of interest. Finally, the number (753) of potential county-level exposures used in our analyses is fairly small relative to the size of the entire spectrum of possible exposome-related variables. Thus, the impact of additional variables as they may be used in subsequent studies is as-yet unknown. Despite these limitations, however, we find the present data adequate, comprehensive enough for exposition and analysis, and sufficiently complex to benefit from graph theoretical methods such as those we describe here that can generate testable hypotheses suitable for deeper investigation.

## 10. Conclusions

We have presented innovative graph theoretical algorithms and described their use in the context of health disparities research. Well-known, standard techniques are able to evaluate only a few potential explanatory variables at a time. Regression, for example, is generally hobbled by 1:10 and 1:20 rules of thumb [51,52]. Modern combinatorial methods such as those we have discussed, however, can unravel and decode unseen signal on an extraordinary scale. In principle, there are no limits other than the statistical significance needed for edgeweight calculations and of course the raw computational power required for graph theoretical operations. Nevertheless, in practice, we generally set thresholds so that subgraph sizes are modest. In the applications discussed here, for example, this typically translates to at most 20 or so variables over more than 3000 counties and county equivalents. This is done mainly to help highlight the most credible latent factors and their inter-relationships, and also to enable and simplify post-processing. Thus, a synergistic fusion of widely divergent but complementary scientific skills can help increase our understanding of population dynamics, pinpoint established and previously unknown relationships and dependencies, address previously-unassailable health disparities questions, and produce a plethora of evidence-based testable hypotheses without preconception.

We emphasize the usefulness of combinatorial “fishing expeditions” such as those we have described here. We are not particularly sanguine about the oft gratuitous use of this particular phrase. Indeed, it is those who appear to miss the point entirely who so often seem to use this term disparagingly. Nevertheless, fishing is in some sense the nature of science and precisely what we desire. A disinclination to cast a broad net is to imply an unwillingness to let go of ingrained and unwavering prejudice and presumption. With the power of mathematical abstraction, highly scalable algorithms and high performance implementations, we can troll through a veritable ocean of unseen relationships. Thus, these tools help us peer deeply and dispassionately into what lurks beneath the surface. Without continuing advances in the discovery process such as these, the rate of scientific progress is restricted by a tradition of hypothesis generation driven only by the mind’s capacity to review and interpret data limited in size, scope and dimensionality. This in turn leads to a natural tendency to conservatism, a lack of new knowledge, and the inherent bias that is born of human frailty.

We also stress the importance of a transdisciplinary team approach. Connecting the dots between divergent disciplines is neither easy nor straightforward. We must recognize that researchers, collaborators and participants may come with vastly differing backgrounds and training, competing expectations for publications and funding, and even different social norms across research communities. From the hieroglyphic-esque notation of mathematics, to the sesquipedalian vocabulary of medicine, to the nuanced interpretations of social science, team members must frequently abandon their comfort zones, set aside their differences, and strive to work together with alacrity in order to have a profound impact on the pace of science. We would be disingenuous not to add that this is very often easier said than done.

## 11. Directions for Future Research

As is frequently the case with the application of new technologies, numerous open questions beckon. We discuss only three:

*Multiple comparisons* pose potential problems in many applications. The methods outlined in this paper are no exception. Such problems may arise, for example, when the probability that a researcher incorrectly concludes that there is at least one statistically significant effect across a set of tests increases with each additional test [53]. Analysts from virtually every social and physical science discipline have encountered situations in which a host of research questions are encountered simultaneously, or many point estimates are being compared. Statistical approaches for dealing with multiple comparisons include relatively simple schemes such as Bonferroni correction, more powerful methods such as weighted Bonferroni procedures [54,55], and approaches that use the false discovery rate to control for the expected proportion of false positives [56,57]. Other recommended procedures include the use of Bayesian multilevel modeling techniques, although this avenue can be challenging for complicated data structures [53]. What approach, or combination of approaches, is most appropriate for health disparities data such as those we have described here?

*Thresholding* is crucial. It determines a graph’s connectivity, degrees, neighborhoods and overall density. The higher is the threshold, the sparser is the graph. Here we have employed multiple thresholds and asked domain scientists, primarily epidemiologists, to evaluate them based on their experience. We have largely been able to automate this process in biological applications through comprehensive studies of *Saccharomyces cerevisiae* (baker’s yeast). This is because biological science has the advantage of model organisms and extensive ontological information. *S. cerevisiae* is without peer in this regard. It is simple and easily reproduced, and serves as a model for all eukaryotic cells. With well-annotated *S. cerevisiae* data, we can evaluate the utility of spectral, graph theoretical and other methods to find inflection points and set logical thresholds. What sort of thresholding testbed can serve similar function in the social sciences, especially in the study of health disparities?

*Validation* has no substitute. Emergent technologies can open many doors, but in retrospect they have sometimes created as many questions as answers. Thus, we must temper our enthusiasm with the knowledge that things are often more complicated than they may seem. A recent example from genetic epidemiology may provide analogy. The field of Genome Wide Association Study (GWAS) began with fanfare, its principal aim to associate DNA variation, such as single-nucleotide polymorphisms, with phenotypical traits, such as disease. There is no pretense of causality. Despite prodigious effort and considerable funding, however, GWAS has in many cases simply failed to deliver the goods. We cite a typical criticism [58]: “GWAS have published hundreds of common variants whose allele frequencies are statistically correlated with various illnesses and traits. However, the vast majority of such variants have no established biological relevance to disease or clinical utility for prognosis or treatment.” With GWAS proponents and detractors so vocal, it remains mired in controversy, all for the lack of validation. Let this not happen in the study of health disparities. What sort of validation earns a seal of approval leading to scientific acceptance?

## Appendix

In the tables that follow, “Label” refers to a vertex label from one of the exemplar graphs. Such a label represents either a paraclique, a latent factor derived from a paraclique, or in rare instances a single variable. “Variables” denotes the number of variables encompassed by that label. “Meaning” briefly describes their composite meaning.

**Table A1:** Prematurity data dictionary.

Label	Variables	Meaning
B POP	3	black proportion and black isolation index
DIAB + OBES	4	diabetes, obesity and inactivity rates
DENTISTS+ INCOME	2	per capita income and dentists private practice
MARRIED	2	percentage married mothers
MCARE DIS+ INCOME	4	medicare enrollment and disabled and white median household income
MOTHER‘S AGE	2	mother age and births to mothers over age 40
POLLUTION+ HEAT	2	pollution heat index and normalized fine particulate matter
PREMATURITY	1	logit of premature singleton birth proportion
STD	2	diagnosed cases of sexually transmitted diseases per population size: chlamydia and gonorrhea
UNINSURED	2	percent less than age 65 without health insurance, females and population total

**Table A2:** Low Black mortality data dictionary.

Label	Variables	Meaning
% DEM	3	percent voters registered as Democrats, voted Democrat in 2004 election and voted Democrat in 2008 election
B EMP + MARRIED	3	rate black males age 16–64 employed, percent blacks and black males married
DIAB + OBES	3	percent residents diabetic and obese
HEAT	5	number of days with temperatures at least 90 degrees, average maximum temperature, average minimum temperature and average land surface temperature
HIGH SES	12	percent black and white adults with at least a four year degree, percent workers employed white collar occupations, median household income and per capita income
HOSP CAP + USE	13	rates per 1000 residents: hospital admissions, inpatient and outpatient surgeries, operating room and hospital beds
MD	8	rates per 1000 residents: primary and specialty care MD’s involved in patient care
MD SPECIALISTS	5	rates per 1000 residents: MD’s including surgical specialists, thoracic surgeons and urologists
SODA$$ + SUNLIGHT	3	average price 12 oz soda and average direct solar radiation in kilojoules per square meter
UNINSURED	3	percent population less than age 65 without health insurance
W HS EDU + BLUE COLLAR	3	percent female adults with only HS education and percent whites and white males employed in blue collar occupations
W INF MORT >1 MILE GROC	3	white infant mortality rate, percent low income residents residing more than 1 mile from grocery and households with no vehicle more than 1 mile from grocery
W L EDU + PHYS INACT	4	percent whites adults with less than HS education and percent residents classified as physically inactive
W POP + SEG	2	percent population non-Hispanic and white isolation index
W POV + PUB ASST	7	percent total and males Medicaid eligible, households on foodstamps and individuals less than age 18 in poverty

**Table A3:** High Black mortality data dictionary.

Label	Variables	Meaning
%W RENTERS	2	percent white households renters
AIR POLLUTION	4	particulate matter pollution, nitrous and nitrogen oxides
B H EDU	3	percent black adults with at least a four year degree
B HH INCOME + MD'S + DINING	5	median black household income, rate per 1000 residents MD’s in private practice and MD’s in other specialties, and percent restaurants full service
B INF MORT + MIDWIVES + OTHER	4	rate per 1000 residents midwives and recreational facilities, black infant mortality rate and percent change in households on foodstamps
GRP HOME + W UNEMP	3	percent population in group homes, white unemployment rate and rate per 1000 residents long term care beds
HEALTH + DISABILITY	4	average subjective health for residents and percent population qualifying for social security disability
HISP WLTH + ETH POP	4	percent population non-Hispanic Asian, percent Hispanic households owning homes valued more than 400 percent median U.S. value, percent population older than age 18, non English speakers and percent population foreign born
MED CARE USE	8	rates per 1000 residents: hospital admissions, inpatient and outpatient surgeries, operating rooms and intensive care beds
MIXED MD SPEC1	13	rates per 1000 residents: total active MD’s, specialists (e.g., neurology, cardiology , surgical specialists and pathologists)
MIXED MD SPEC2	7	rates per 1000 residents: office based MD’s, pediatricians, internists, psychiatrists, child psychiatrists and white per capita income
MIXED MD SPEC3	4	rates per 1000: MD diagnostic radiologists, orthopedic surgeons, gastroenterologists and anesthesiologists
PI POP WLTH	3	percent population non-Hispanic Pacific Islanders and percent black and white households owning homes valued more than 400 percent median U.S. value
W CLG EDU	2	percent white adults with at least a four year degree
W HS EDU + BLUE COLLAR	3	percent total white and white male workers employed in blue collar occupations and percent adult males with HS education only
W L EDU	3	percent white adults with less than HS education
W MARRIED	2	percent white and white females married
W + ALL HH INC + DENTISTS	4	white and total median household income and rate per 1000 residents active dentists

**Table A4:** Black male high lung cancer data dictionary.

Label	Variables	Meaning
%DEM	3	percent voters registered or voted Democratic 2004–2008
%REP	3	percent voters registered or voted Republican 2004–2008
AGE + AGE DISABLED	8	median age female, white non-Hispanic, non-Hispanic male, percent medicare enrolled aged, disabled, percent white males age 65 plus
B L EDU	4	rate black low education, education less than HS in blacks, education low black female and male
B POP + SEG + LBW	4	black isolation index 2000, low birth weight, percent non-Hispanic blacks 2008, percent black/African-American population
DEP CHILD POV	9	rate unmarried, percent free lunch, poverty less than age 18, foodstamp recipients; medicaid eligible female/male/total, poverty rate under age 18
DIAB + OBES + INACTIV	4	percent diabetes in adults, percent obese adults, adjusted percent inactive, age-adjusted obesity 2009
ELD POP	3	percent residents over age 65, eligible for Medicare and Medicare disability
HOSP BEDS	3	rate hospital beds: licensed, community hospital, hospital, licensed short term, short term, total inpatient
HOSP SURG	3	rate operating rooms, intensive care beds, medical-surgical adult beds
LOW LIT MI GROC	3	percent low literacy, percent low income over 10 miles to store 2006, percent households no car over 1 mile to store 2006, percent low income more than 10 miles to store 2006
MED CARE USE 1	5	rates per 1000 residents: outpatient visits, outpatient and total surgeries, medical-surgical pediatric beds
MED CARE USE 2	3	rates per 1000 residents: community hospital admission, hospital admission, short term hospital admission
MED SERV MIXED	4	rate total MD’s family medicine, cardiology intensive care beds, neonatal intensive beds, total thoracic surgery beds
MIXED MD SPEC1	27	rate mixed medical specialties
MIXED MD SPEC2	6	rate per 1000 residents: MDs, gastroenterologists, ob-gynecologists, opthomologists and otoloryngologists

**Table A5:** White male high lung cancer data dictionary.

Label	Variables	Meaning
%DEM	3	percent voters registered or voted Democratic 2004–2008
%REP	3	percent voters registered or voted Republican 2004–2008
B POP + LBW	6	black isolation index 2000, low birth weight, percent non-Hispanic blacks 2008, percent black/African-American population, unmarried and very low birth weight
B POP + SEG	3	black isolation index 2000, percent non-Hispanic blacks 2008, percent black/African-American population
HOSP BEDS	5	rate hospital beds: licensed, community hospital, hospital, licensed short term, short term and total inpatient
MD SPEC + NEONATAL	3	rate total neurosurgery, neonatal intensive beds and total thoracic surgery total patient care
MED CARE USE 1	6	rate hospital admission: community hospital, hospital, short term hospital. surgical operations: inpatient, outpatient, total
MIXED MD SPEC1	10	rate MDs in patient care 2005, office based on non-office based and some specialties
MIXED MD SPEC3	3	rate MDs hospital residents, pathologists and psychologists
PERS DEPRIV	3	percent persistent poverty and child poverty.
POV + PUB ASST + UNEQUAL	7	percent free lunch, foodstamp recipients, medicaid eligible and poverty rate
W DEPRIV + DISABILITY	9	percent households in poverty, lower than HS education and medicare disability enrollment
W INC	3	household income white
W POP	3	white isolation index 2000, percent non-Hispanic whites 2008, percent black/African-American population
